# Multifocal gastrointestinal stromal tumor with osseous metaplasia: a case report

**DOI:** 10.1186/s13256-023-04262-9

**Published:** 2023-12-15

**Authors:** Maryam Abdullah Al Saleem, Nida Mirzaman Khan, Tarek Mohammad ElSharkawy

**Affiliations:** Department of Pathology, King Fahd Hospital of University, College of Medicine, Imam Abdulrahman Bin Faisal University, Khobar, Saudi Arabia

**Keywords:** Gastrointestinal stromal tumor, Osseous metaplasia, Osseous differentiation, Metaplasia, *PDGFA*, Case report

## Abstract

**Background:**

Gastrointestinal stromal tumor is considered the most common mesenchymal neoplasm of the gastrointestinal tract. The majority of gastrointestinal stromal tumor cases are located in the stomach and usually affects older adults. Most of gastrointestinal stromal tumor cases are sporadic; however, few have a syndromic association, including Carney triad, Carney–Stratakis syndrome, familial gastrointestinal stromal tumor syndrome, and neurofibromatosis type 1.

**Case presentation:**

Herein, we report a rare case of a 54-year-old Middle-Eastern female with multifocal gastrointestinal stromal tumor mixed type (epithelioid and spindle cell type) with osseous metaplasia. Fluoresce *in situ* hybridization analysis of platelet-derived growth factor receptor alpha revealed deletion in 42% of the tumor cells studied. Interestingly, next generation sequencing revealed platelet-derived growth factor receptor alpha exon 12 mutation (p.Y555C) and exon 14 mutation (p.N659Y).

**Conclusions:**

In conclusion, osseous metaplasia in GIST is a very rare event and only few cases are reported in the literature. The number of reported cases is inadequate to confirm the pathogenesis and the prognosis.

## Background

Gastrointestinal stromal tumor (GIST) is a mesenchymal neoplasm of the gastrointestinal tract. Mesenchymal neoplasms of the gastrointestinal tract are rare, and GIST is the most common accounting for 80% [1]. GIST rises from interstitial cells of Cajal within the myenteric plexus of the muscularis propria that is distributed through the esophagus to the internal anal sphincter [2, 3]. Usually, GIST affects older adults, and it is considered very rare in young adults and pediatric population, with the exception of GISTs associated with syndromes [4]. There is no sex predilection with the exception of succinate dehydrogenase-deficient GIST, which has a female predominance [4, 5]. Most of the GIST cases are sporadic; however, few have a syndromic association including Carney triad, Carney–Stratakis syndrome, familial GIST syndrome, and neurofibromatosis type 1 (NF1) [4]. The majority of GIST cases are located in the stomach (60%), jejunum and ileum (30%), duodenum (4–5%), rectum (4%), colon and appendix (1–2%), and esophagus (< 1%) [4, 6]. However, the primary arising of GIST in extraintestinal locations, such as the mesentery, omentum, or retroperitoneum, is very rare.

GIST may present as gastrointestinal bleeding, abdominal pain, and intestinal obstruction, or it might be incidentally discovered. Radiologically, smaller GISTs appear as a well circumscribed homogeneous mass [7]. Larger GIST could be well or ill-defined, heterogeneous mass with peripheral enhancement with areas of hemorrhage, necrosis, or cystic degeneration [7]. Macroscopically, GIST is a well circumscribed intramural lesion with a fleshy white-tan surface and may show areas of necrosis, hemorrhage, or cystic degeneration [8]. Microscopically, GIST is divided by morphology into three types: spindle (70%), epithelioid (20%), and mixed (10%) [9]. The most common type is composed of spindle cells with indistinct cell borders, ovoid nuclei, and inconspicuous nucleoli with faint eosinophilic cytoplasms [8, 9]. Paranuclear vacuoles are commonly seen in gastric GIST [8]. The epithelioid type is composed of round cells with clear to eosinophilic cytoplasm organized in nests, sheets, or rarely in cords [8, 9]. The mixed type is composed of cells with both spindle and epithelioid morphology [8, 9]. The majority of GISTs have *KIT* mutations, which can be treated by tyrosine kinase inhibitors such as imatinib mesylate for unresectable or metastatic tumors. The risk stratification for untreated GISTs with imatinib is based on anatomic site, size, and mitotic count [10].

## Case presentation

A 54-year-old Middle-Eastern female, a known case of diabetes mellitus, dyslipidemia, and hypertension, presented to King Fahad Hospital of Imam Abdulrahman Bin Faisal University in Khobar complaining of dizziness for 1 year associated with nausea, vomiting, and diarrhea. The vomiting was food content and related to food intake. Patient’s past surgical history included open appendectomy 20 years ago, open cholecystectomy 15 years ago, and incisional hernia repair 5 years ago.

On physical examination, the patient was conscious, alert, and oriented to time, place, and person. She was afebrile, normotensive, had a normal heart rate, and her abdomen was soft and lax.

Endoscopy revealed esophagus with more than one mucosal break greater than 5 ml without connection in between mucosal folds (type B gastroesophageal reflux disease). The stomach showed mild erythema and small subepithelial lesion near incisura angularis at 12 o’clock. The pylorus, duodenal cap, and second part of the duodenum were all normal. An endoscopic biopsy was taken from the stomach and epithelial lesions were diagnosed as inactive mild chronic gastritis with a negative Warthin starry stain for *Helicobacter pylori* organisms.

The patient was referred to the radiology department for further investigations. Computed tomography (CT) scan revealed multiple well-defined exophytic lesions seen along the greater curvature of the stomach. These lesions demonstrated low signal to isosignal on T1-weighted images and bright signal on fat saturated T2-weighted images with intense enhancement on post-contrast administration, and mild restriction on diffusion-weighted images. The largest mass measured 2 cm × 0.8 cm and was abutting the liver anteriorly, but with no signs of invasion. The remainder of the lesions had an average maximum diameter of 1.2 cm. The radiological impression at the time of examination was gastrointestinal stromal tumor (GIST). An alternative possibility was a neurogenic tumor. The patient was scheduled for a diagnostic and therapeutic laparoscopic partial vertical gastrectomy. Operative findings were multiple lesions along the greater curvature and the largest lesion was subhepatically located with adhesion. Also, a large incisional hernia with incarcerated omentum was seen.

**Fig. 1:**
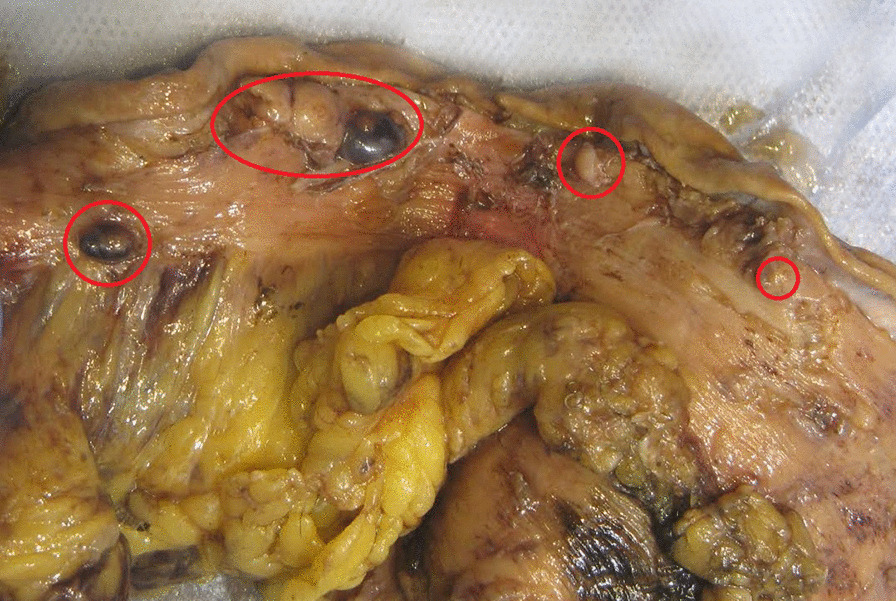
Gross appearance of the small firm serosal nodules along the greater curvature. (Four small firm serosal nodules (Red circle) were identified along the greater curvature)

A 10% buffered formalin-fixed partial vertical gastrectomy was sent for histopathological examination. Macroscopically, it consisted of a partial vertical gastrectomy specimen, measuring 20.4 cm × 4.3 cm × 2.9 cm with 0.8 cm maximum wall thickness, and an attached 12.5 cm × 17 cm × 0.8 cm perigasteric fibrofatty tissue. Four small firm serosal nodules were identified along the greater curvature, measuring 1 cm × 0.6 cm × 0.4 cm, 0.5 cm × 0.4 cm × 0.2 cm, 0.5 cm × 0.3 cm × 0.3 cm, and 0.4 cm × 0.3 cm × 0.3 cm (Fig. 1). All the nodules had a white-tan cut surface, and were away from the proximal and distal margins by at least 4.5 cm. Also, one gray-tan lymph node measuring 3.8 cm × 3.5 cm × 1 cm within the perigastric fibrofatty tissue was identified.

**Fig. 2:**
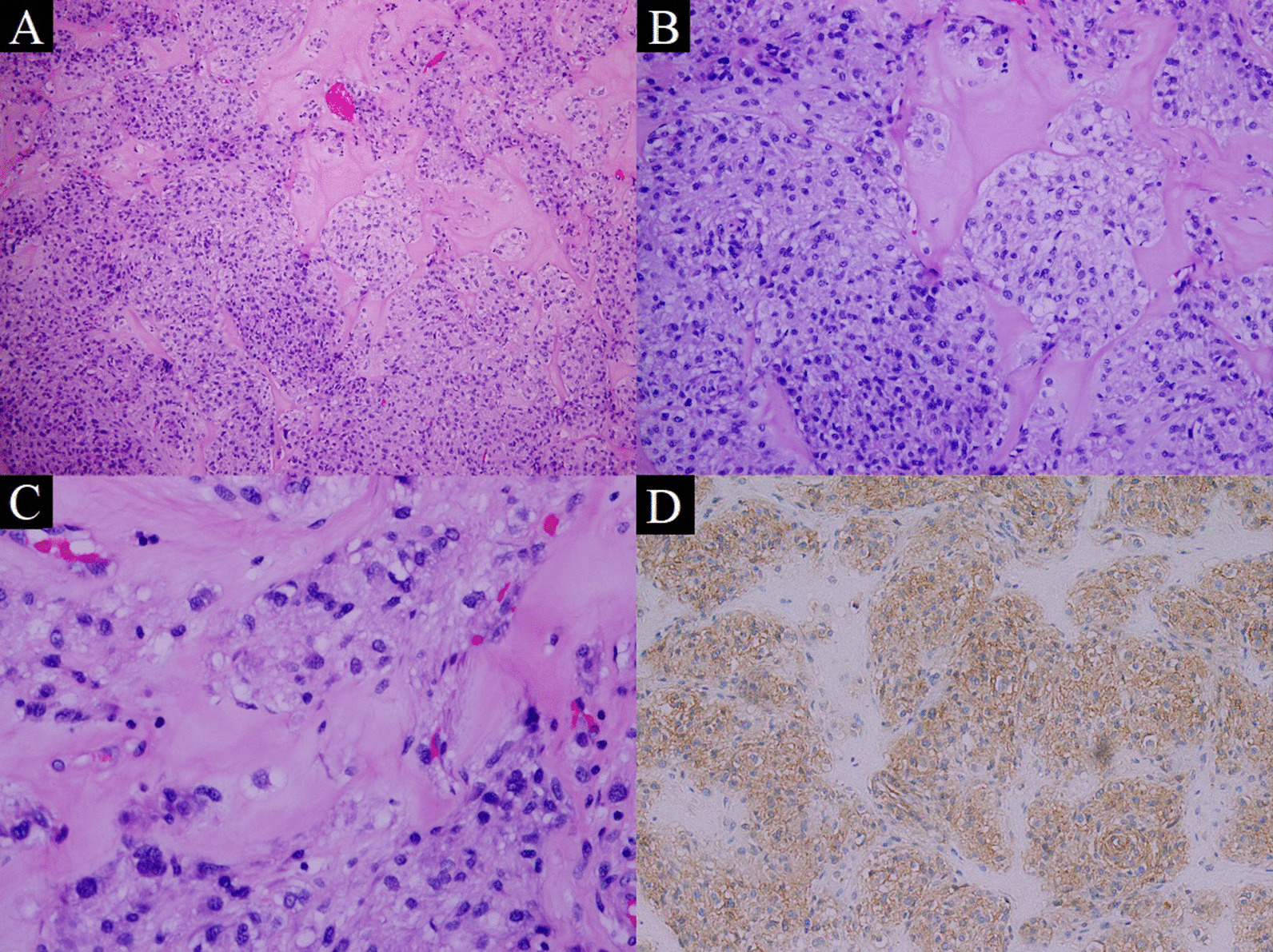
Hematoxylin and eosin (H&E) stained sections show intermixed populations of spindle and epithelioid cells with osseous metaplasia and bone formation (**A**–**C**). Immunohistochemical stain (DOG1) showing lymph node positive for metastatic GIST (**D**)

Microscopically, hematoxylin and eosin (H&E) stained sections of the grossly identifed four nodules revealed a similar histological features of a cellular well-circumscribed lesion without capsule. Higher magnification revealed intermixed populations of cells, consisting of bland spindle cells in a syncytial pattern and epithelioid cells with plump eosinophilic cytoplasm. The nuclei were bland, elongated to round in shape with a mitotic rate of 2/5 mm^2^. An additional interesting finding found in the first and last nodules was osseous metaplasia and bone formation (Fig. 2A–C). A single lymph node identified positive for metastasis accompanied with osseous metaplasia, bone formation, and tumor necrosis that ranged in between 5% and 10%

Differential diagnosis based on morphological features were gastrointestinal stromal tumor (GIST), perivascular epithelioid cell tumor (PEComas), neuroendocrine tumor, leiomyoma, schwannoma, desmoid tumor, adenocarcinomas, and glomus tumor.

**Fig. 3:**
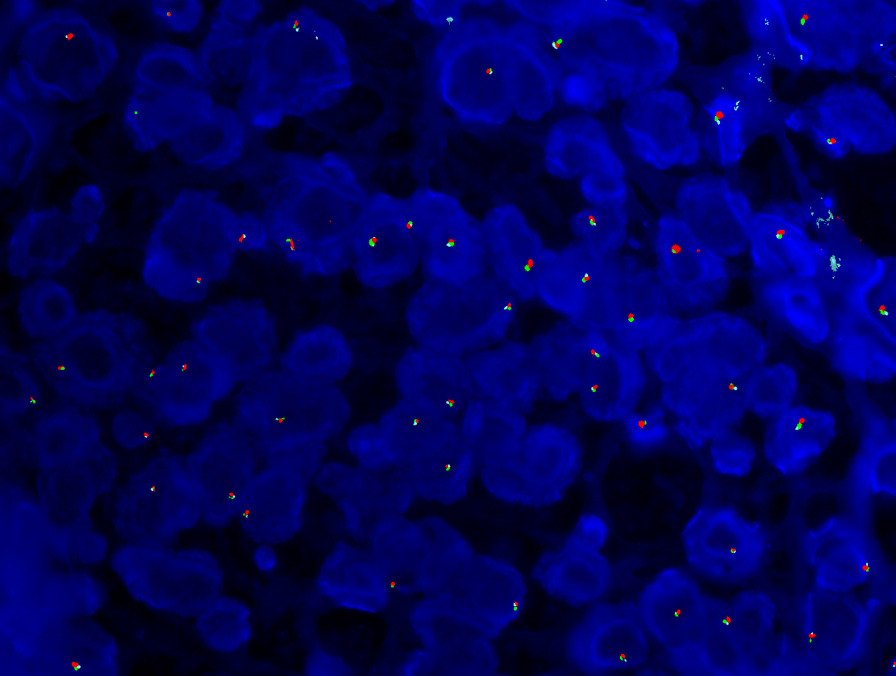
FISH analysis using *PDGRFA* probe. The tumor cells show one red on green signal, indicative of deletion of one copy of *PDGRFA*

The immunohistochemical studies for all nodules and metastasis were positive for c-kit (CD117), discovered on GIST 1 (DOG1), and CD34, supporting GIST diagnosis (Fig. 2D). Desmin, β-catenin, cytokeratin 7 (CK7), cytokeratin 20 (CK20), smooth muscle actin (SMA), epithelial membrane antigen (EMA), S100 protein, melanoma antigen (melan A), and human melanoma black (HMB45) were all negative precluding other diagnosis. Fluoresce in situ hybridization (FISH) analysis of *PDGFRA* done for metastasis showed deletion in 84 out of 200 (42%) interphase nuclei scored (Fig. 3). Next generation sequencing done for metastasis revealed *PDGFRA* exon 12 mutation (p.Y555C) and exon 14 mutation (p.N659Y), suggesting a good response to tyrosine kinase inhibitor.

Based on the above findings, the diagnosis of multifocal gastrointestinal stromal tumor, mixed type (epithelioid and spindle cell type), with osseous metaplasia. Although the calculated risk for metastasis and or death (risk of progressive disease) was very low (1.9%) [6]. The tumor metastasized to the lymph node raising the Pathologic Stage Classification (AJCC, 8th edition) to pT1(m)N1. The patient was referred to oncology clinic to start tyrosine kinase inhibitor (imatinib) 400 mg per day. Follow-up of the patient with abdominal CT scan after 2 years revealed interval partial gastric resection without evidence of local recurrence of the disease.

## Discussion

Gastrointestinal stromal tumor (GIST) with osseous metaplasia was first described by Deepa et al. as a rare stromal event in a photomicrograph in their review [10]. Giorlandino et al. reported one case of a 60-year-old man affected by a GIST with benign osseous metaplasia and mature bone formation presented with abdominal pain [10]. Another case of a 65-year-old male affected by a GIST with mature bone formation, osseous metaplasia, and calcification presented with abdominal pain, as reported by Haneen et al. [11]. Finally, Riveros Gilardi et al. described a photomicrograph of GIST with osseous metaplasia as the fourth case reported in the literature [12].

There are many theories about the pathogenesis of osseous metaplasia, but the most recent morphoproteomic supported study by Richard et al. states that osseous metaplasia originates from the transition of stromal pluripotent cells into an osteoblast under the influence of growth factors secreted by the tumor cells [13]. Growth factors that might be involved in the osseous metaplasia process include alkaline phosphatase, bone morphogenetic protein (BMP), GLI family zinc finger 2 (GLI2), transforming growth factor–beta (TGF-β), and α-Smooth muscle actin (α-SMA) [13, 14]. However, integration of these factors and the pathogenesis is not clear. Another theory is that chronic inflammation has a role as the fibroblast can be transformed to any mesodermal-derived cells, including osteoblasts [15]. Finally, there is a theory for imatinib-treated GISTs. Narasimhan et al. observed in his study that imatinib-treated GISTs showed muscle differentiation due to upregulation of genes involved in it [16]. Therefore, raise the possibility of osseous differentiation as a response for imatinib [11, 17]. 

Approximately, 75% of sporadic GIST are associated with *KIT* oncogenic mutations, which constitutively activate the receptor tyrosine kinase and *KIT*-dependent signaling pathways that end up in increasing the cell proliferation and apoptosis [18]. Mutations most commonly occur in *KIT* exon 11 (75%) that range from in-frame deletions, to insertions, to point mutations, or substitutions [9, 18]. Around 10% of GISTs display *KIT* mutation in exon 9, more frequently seen in GIST arising in the intestine [9]. However, exon 13 and exon 17 rarely mutated [9]. A minor subsets of GISTs (10%) showed mutations in *PDGFRA*, mainly involving exon 18, exon 12, or exon 14. After *KIT* mutation, *PDGRFA* is considered the second most common mutation in GIST [19]. *PDGFRA*-mutant GIST is commonly associated with epithelioid morphology [9]. *KIT* and *PDGFRA* mutations are mutually exclusive in GIST [9]. SDH-deficient GIST is associated with germline mutations in *SDHA*, *SDHB*, *SDHC*, or *SDHD*, resulting in succinate accumulation [18]. This, in turn, increases the transcription of *HIF1α*-regulated genes and decreases DNA methylation [18]. Another subset of GIST revealed *BRAF* (V600E) mutations with the tendency to be located in the small intestine [4].

However, neoplasms with osseous metaplasia have a similar genetic profile to corresponding ones without osseous metaplasia [20]. Due to limited numbers of GISTs with osseous metaplasia, it is not known if it is associated with a certain genetic aberration, if it has a tendency toward a specific location, or if it has any prognostic impact.

## Conclusions

In conclusion, osseous metaplasia in GIST is a very rare event and only few cases are reported in the literature. The number of reported cases is inadequate to confirm the pathogenesis nor the prognosis. More studies on reported cases of GIST with osseous metaplasia may further help to expand our knowledge and understand its pathogenesis, clinical significance, and prognosis.

## Data Availability

Data sharing is not applicable to this article as no datasets were generated or analyzed during the current study.
